# Radiographic correlates of hallux valgus severity in older people

**DOI:** 10.1186/1757-1146-4-S1-O14

**Published:** 2011-05-20

**Authors:** Paul R D’Arcangelo, Karl B Landorf, Shannon E Munteanu, Gerard V Zammit, Hylton B Menz

**Affiliations:** 1 Department of Podiatry, Faculty of Health Sciences, La Trobe University, Bundoora, Victoria 3086, Australia; 2 Musculoskeletal Research Centre, Faculty of Health Sciences, La Trobe University, Bundoora, Victoria 3086, Australia

## Background

The severity of hallux valgus is easily appreciated by its clinical appearance, however x-ray measurements are also frequently used to evaluate the condition, particularly if surgery is being considered. There have been few large studies that have assessed the validity of these x-ray observations across a wide spectrum of the deformity. In addition, no studies have specifically focused on older people where the progression of the disorder has largely ceased. Therefore, this study aimed to explore relationships between relevant x-ray observations with respect to hallux valgus severity in older people.

## Methods

This study utilised 402 x-rays of 201 participants (74 men and 127 women) aged 65 to 94 years. All participants were graded using the Manchester Scale - a simple, validated system to grade the severity of hallux valgus - prior to radiographic assessment. A total of 19 hallux valgus-related x-ray observations were performed on each set of x-rays. These measurements were then correlated with the Manchester Scale scores.

## Results

Strong, positive correlations were identified between the severity of hallux valgus and the hallux abductus angle, the proximal articular set angle, the sesamoid position and congruency of the first metatarsophalangeal joint. As hallux valgus severity increased, so did the frequency of radiographic osteoarthritis of the first metatarsophalangeal joint and a round first metatarsal head. A strong linear relationship between increased relative length of the first metatarsal and increased severity of hallux valgus was also observed.

## Conclusions

Strong associations are evident between the clinical appearance of hallux valgus and a number of hallux valgus-related x-ray observations indicative of structural deformity and joint degeneration. As it is unlikely that metatarsal length increases as a result of hallux valgus deformity, increased length of the first metatarsal relative to the second metatarsal may be a contributing factor to the development and/or progression of hallux valgus.

